# Prevalence and characteristics of patients with heart failure needing palliative care

**DOI:** 10.1186/s12904-021-00850-y

**Published:** 2021-12-02

**Authors:** Luisa Fernanda Arenas Ochoa, Valentina González-Jaramillo, Clara Saldarriaga, Mariantonia Lemos, Alicia Krikorian, John Jairo Vargas, Xavier Gómez-Batiste, Nathalia Gonzalez-Jaramillo, Steffen Eychmüller

**Affiliations:** 1 Pain and Palliative Care Group, School of Health Sciences, Universidad Pontificia Bolivariana, Medellín, Colombia; 2 Department of Palliative Care, Clínica Cardio VID, Medellín, Colombia; 3 Institute of Social and Preventive Medicine (ISPM), University of Bern, Bern, Switzerland; 4 Graduate School for Health Sciences, University of Bern, Bern, Switzerland; 5 Department of Cardiology, Clínica Cardio VID, Medellín, Colombia; 6 Cardiology Department, Universidad de Antioquia, Medellín, Colombia; 7 Department of Psychology, School of Humanities, Universidad EAFIT, Medellín, Colombia; 8 Institute of Cancerology, Clínica Las Américas, Medellin, Colombia; 9 Chair Qualy Palliative Care, Faculty Medicine, University of Vic/Central of Catalonia, Barcelona, Spain; 10 University Center for Palliative Care, Inselspital University Hospital Bern, University of Bern, Bern, Switzerland

**Keywords:** Heart failure, Palliative care, Needs assessment, Prognosis, Patient-centered care, Health services needs and demands

## Abstract

**Background:**

Few hospitals and heart failure (HF) clinics offer concurrent palliative care (PC) together with life-prolonging therapies. To know the prevalence of patients in HF clinics needing PC and useful tools to recognize them are the first steps to extending PC in those settings. However, it is still unknown whether tools commonly used to identify patients with HF needing PC can correctly distinguish them. Two systematic reviews found that the NECesidades PALiativas (NECPAL) tool was one of the two most commonly used tools to asses PC needs in HF patients. Therefore, we assessed 1) the prevalence of PC needs in HF clinics according to the NECPAL tool, and 2) the characteristics of the patients identified as having PC; mainly, their quality of life (QoL), symptom burden, and psychosocial problems.

**Methods:**

This cross-sectional study was conducted at two HF clinics in Colombia. We assessed the prevalence of PC in the overall sample and in subgroups according to clinical and demographic variables. We assessed QoL, symptom burden, and psychosocial problems using the 12-Item Short-Form Health Survey (SF-12), the Kansas City Cardiomyopathy Questionnaire (KCCQ) and the Edmonton Symptom Assessment System (ESAS). We compared the results of these tools between patients identified as having PC needs (+NECPAL) and patients identified as not having PC needs (–NECPAL).

**Results:**

Among the 178 patients, 78 (44%) had PC needs. The prevalence of PC needs was twice as nigh in patients NYHA III/IV as in patients NYHA I/II and almost twice as high in patients older than 70 years as in patients younger than 70 years. Compared to –NECPAL patients, +NECPAL patients had worse QoL, more severe shortness of breath, tiredness, drowsiness, and pain, and more psychosocial problems.

**Conclusion:**

The prevalence of PC needs in outpatient HF clinics is high and is even higher in older patients and in patients at more advanced NYHA stages. Compared to patients identified as not having PC needs, patients identified as having PC needs have worse QoL, more severe symptoms, and greater psychosocial problems. Including a PC provider in the multidisciplinary team of HF clinics may help to assess and cover these needs.

**Supplementary Information:**

The online version contains supplementary material available at 10.1186/s12904-021-00850-y.

## Background

Through the heart failure (HF) trajectory, patients have a wide range of physical and psychological symptoms that affects their quality of life (QoL) [1]. According to the World Health Organization (WHO), palliative care (PC) is an approach that aims to improve the QoL of patients and families facing challenges associated with a chronic condition, whether physical, psychological, social, or spiritual [2]. Despite current recommendations to incorporate a PC approach into the standard care of patients in advanced stages of HF [1, 3–6], important gaps have been identified in its delivery [7]. Although widely used for oncological patients, few hospitals and HF clinics offer concurrent PC together with life-prolonging therapies. To know the prevalence of patients in HF clinics needing PC and useful tools to recognize them are the first steps to extending PC in those settings. However, it is still unknown whether tools commonly used to identify patients with HF needing PC can correctly distinguish them. The NECesidades PALiativas (NECPAL) tool was created to identify in clinical practice patients with chronic disease and a limited life expectancy who might benefit from PC [8]. The tool has been widely used in clinical practice in different countries and is currently available in several languages [8–11]. Two recent systematic reviews found that, among the studies assessing PC needs in patients with HF, the NECPAL tool was one of the two most commonly used screening tools [12, 13].

## Methods

This study was conducted and reported in accordance with the STROBE guidelines (Supplementary Material 1) [14].

### Aim

In this study, we assessed 1) the prevalence of PC needs in outpatient HF clinics according to the NECPAL tool, and 2) the characteristics of the patients identified as having PC needs; mainly, their health-related QoL, symptom burden, and psychosocial problems, assessed using the 12-Item Short-Form (version 2) Health Survey (SF-12) [15], the Kansas City Cardiomyopathy Questionnaire (KCCQ) [16], and the Edmonton Symptom Assessment System (ESAS) [17].

### Study design and setting

This cross-sectional study was conducted at two HF clinics. Both clinics are part of tertiary care institutions in Medellin (Colombia) that are referral centers for patients with cardiovascular disease. They offer comprehensive, multidisciplinary care that includes clinical follow-up by HF cardiologists, nursing education and telephone follow-up, cardiac rehabilitation, and a psychoeducational program for both patients and their families.

### Participant selection

We invited consecutive eligible patients to participate in the study. Patients were eligible to participate if they were ≥ 18 years old and were already enrolled in the HF clinic. New patients in HF clinics may not be receiving optimal treatment according to clinical guidelines; therefore, the first consultations are fundamental for adjusting the treatment if necessary. An inclusion criterion was to have attended at least two appointments at the HF clinic before enrolling in the study so that the cardiologist could evaluate the patient under optimal treatment and rule out inadequate management as a cause of symptoms. Those patients who were identified by the treating cardiologist or research assistant as having cognitive problems in understanding or answering the questions on the instruments were excluded from the study.

### Ethical aspects

All the participants provided informed consent before the enrolment. The study was carried out according to the guidelines of the Declaration of Helsinki [18] and was granted ethical approval.

## Data collection

Enrollment occurred between October 2017 and November 2018. Participants were asked to fill out three instruments: the SF-12 [15], the KCCQ [16], and the ESAS [17]. A research assistant was available to support patients in case of queries when answering the instruments. A researcher scored the answers from the SF-12 and the KCCQ instruments. The attending cardiologist filled out the NECPAL tool [8].

## Sociodemographic and clinical characteristics

To describe the population included in the study, we obtained patient sociodemographic characteristics and clinical variables from electronic medical records. Sociodemographic characteristics included age, sex, marital status, and religious affiliation. Clinical variables included left ventricular ejection fraction (LVEF), number of hospitalizations in the last year, presence of an implantable cardiac device, functional class according to the New York Heart Association (NYHA), comorbidities, and medications. Comorbidities included atrial fibrillation, type 2 diabetes mellitus, kidney disease, lung disease, coronary artery disease, obstructive sleep apnea, and hypothyroidism. Medications included angiotensin-converting enzyme inhibitors, beta-blockers, and angiotensin receptor blockers.

## The NECPAL CCOMS-ICO (NECPAL) tool

The NECPAL CCOMS-ICO© tool (in Spanish, NECesidades PALiativas; in English, Palliative Needs) [8] consists of four blocks of questions 1) The surprise question, which aims to identify patients with limited life expectancy, is a reflexive question health care providers ask themselves about the patient´s prognosis: would I be surprised if this patient dies within the next 12 months? A surprise question is positive if the health care provider's answer is no, I would not be surprised. In this event, the health care provider continues assessing the other three blocks. 2) Request for PC care by health professionals or the patient/family, 3) clinical markers of health status and frailty that mainly focus upon nutrition, functional status, emotional distress, and comorbidities, and 4) disease-specific clinical prognostic markers. Patients with a positive NECPAL (+NECPAL) are those in need of PC. A patient is considered to have a +NECPAL if block 1, the surprise question, is positive and at least one of the other three blocks is positive. Otherwise, patients have a negative NECPAL (–NECPAL) and, according to the tool, do not need PC.

## The 12-Item Short-Form Health Survey

This 12-item SF-12 is a subset of the SF-36 Survey, which is one of the most widely used instruments to evaluate health-related QoL. We used a Spanish version of the survey that had been validated previously and shown good internal consistency with a Cronbach´s alpha of 0.7 at its validation [15]. The survey score ranges from 0 to 100, with higher scores indicating better QoL. It assesses subjects´ perception of their health and their limitations in activities of daily life. The 12 items are grouped in eight subscales from which two summary measures derive. The physical summary is derived from the subscales physical function, physical role, body pain, and general health, and the mental summary results from the subscales vitality, social function, emotional role, and mental health.

## The Edmonton Symptom Assessment System (ESAS)

This tool evaluates the presence and severity of common symptoms in the PC context. We used a previously validated Spanish version of the tool that assess 10 symptoms: pain, tiredness, drowsiness, nausea, lack of appetite, shortness of breath, depression, anxiety, sleep disturbances, and wellbeing, and an additional symptom that the patient is free to name [17]. The scores range from 0 to 10, with higher scores indicating worse severity of the item assessed and 0 indicating its absence.

## The Kansas City Cardiomyopathy Questionnaire (KCCQ)

This questionnaire was designed to quantify the health status of patients with heart failure and its impact on their QoL. We used a previously validated Spanish version of the questionnaire that consists of 23 items that are grouped in seven dimensions: physical limitations; stability, frequency, and severity of symptoms; self-efficacy; QoL; and social limitations. In addition, two summary scores are calculated: the clinical summary and the general summary [16]. The score ranges from 0 to 100, with higher scores indicating better QoL.

## Data analysis

Categorical data were summarized as count (%). Continuous variables were summarized as mean (SD) if they had a normal distribution or as median (interquartile ranges [IQR]) if they had a non-normal distribution. We assessed normality using Q-Q plots.

## Sample size calculation

For the sample size calculation, we assumed a prevalence of PC needs in HF programs of 50% due to the lack of information in the literature. With an expected proportion of PC need of 50%, a 95% confidence level, and a precision of 5%, the required sample size was 178 patients.

## Prevalence of palliative care needs according to NECPAL tool

The prevalence of patients with PC needs was calculated using the proportion of patients with +NECPAL (numerator) out of the total number of patients included in the study (denominator). We assessed the prevalence within age groups, sex, NYHA functional class, and LVEF classification. To do this, we created a categorical variable for age according to the median, and another for LVEF as follows: reduced if LVEF was ≤40%, mildly reduced if LVEF was between 41% and 49%, and preserved with LVEF ≥50% [19]. We compared prevalence between categories with a chi-square test.

## Characteristics of the patients with heart failure identified as having palliative care needs

Palliative care aims to improve QoL by assessing and treating pain and other physical and psychosocial problems [20]. Therefore, we compared NECPAL groups (–NECPAL and +NECPAL) according to health-related QoL, pain, other physical problems, and psychosocial problems. We evaluated these characteristics using the F-12, the KCCQ, and the ESAS.

We used the Mann-Whitney U test to compare scores between groups. All analyses were performed using STATA release 15 (Stata Corp, College Station, Texas).

### Health-related quality of life

We used physical and mental summaries of the SF-12, and the QoL dimension from the KCCQ to assess health-related QoL.

### Pain

We used the subscale body pain of the SF-12 and extracted the pain score from the ESAS.

### Other physical problems

Other physical problems included tiredness, drowsiness, nausea, lack of appetite, shortness of breath, and sleep disturbances. These were extracted from the ESAS.

### Psychological problems

To assess psychological problems, we used the emotional role and mental health subscales of the SF-12 and the self-reported depression and anxiety scores from the ESAS.

### Social problems

The subscale social function was extracted from the SF-12 and the dimension social limitation from the KCCQ.

## Results

### General characteristics of the participants included in the study

**Fig. 1:**
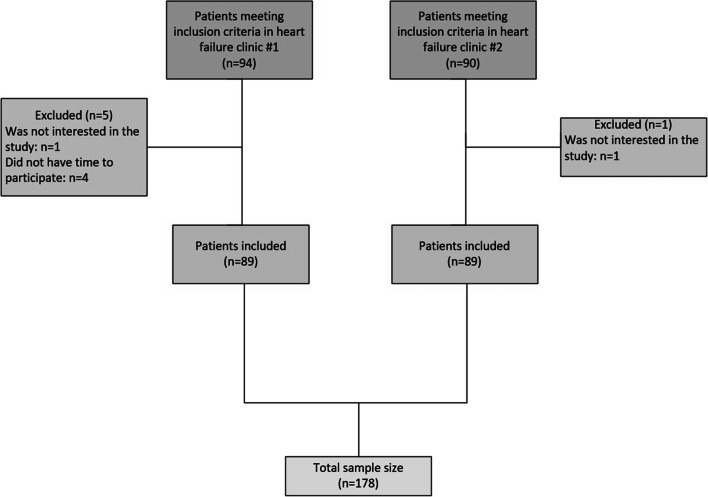
Flowchart of the participants included in the study

Of the 184 patients who met the inclusion criteria, 178 accepted participation, 89 from each HF clinic (Fig. 1).

Table 1 shows the baseline demographic and clinical characteristics of the 178 participants. The population had a median age of 70 (58-77) and 99 (56%) were male. The majority (86, 48%) were classified as NYHA class II, followed by NYHA I (57, 32%), and NYHA III (34, 19%). Only one patient was classified as NYHA IV. The majority of the patients (118, 66%) had a reduced LVEF. Among the remainder, 29 (16%) had a mildly reduced LVEF, and 31 (18%) a preserved LVEF. Among the 73 patients with an implantable cardiac device, 45 (62%) had an implantable cardioverter defibrillator.

**Table 1:** Clinical and demographic characteristics of patients included in the study

	Patients included in the study (n=178)
**Sociodemographic characteristics**	
Age in years	70 (58-77)
Sex	
Men	99 (55.6)
Women	79 (44.4)
Marital status	
Single	25 (14.0%)
Married	94 (52.8%)
Separated or divorced	11 (6.2%)
Widow(er)	48 (27.0%)
Religious affiliation	
Yes	177 (99.4%)
No	1 (0.6%)
**Clinical variables**	
LVEF (%)	32 (25-45)
Classification according to the LVEF	
HFrEF	118 (66%)
HFmrEF	29 (16%)
HFpEF	31 (18%)
Hospitalizations in the last year	
0	1 (0.6%)
1	46 (25.8%
2	109 (61.2%)
>2	22 (12.4%)
Presence of implantable cardiac device	
Yes	73 (41.0%)
No	105 (59.0%)
NYHA functional class	
I	57 (32.0%)
II	86 (48.3%)
III	34 (19.1%)
IV	1 (0.6%)
Comorbidities	
Atrial fibrillation	53 (29.8%)
Type 2 diabetes mellitus	56 (31.5%)
Chronic kidney disease	86 (48.3%)
Chronic obstructive pulmonary disease	25 (14.0%)
Coronary artery disease	66 (37.1)
Obstructive sleep apnea	13 (7.3%)
Medication	
Beta-blockers	168 (94.4%)
ACE inhibitors	140 (78.7%)
ARBs	134 (75.3%)

Data presented as amount of patients and (%) for categorical data, or as median and (IQR) for continuous data

LVEF: left ventricular ejection fraction; HFrEF: heart failure with reduced ejection fraction; HFmrEF: heart failure with mildly reduced ejection fraction; HFpEF: heart failure with preserved ejection fraction; ACE: angiotensin-converting-enzyme; ARBs: angiotensin receptor blockers

Patients from the two HF clinics had similar characteristics; they were mainly patients with reduced ejection fraction, male, and at NYHA II. The prevalence of hypertension, coronary disease, diabetes mellitus, COPD, and chronic kidney disease, and use of implantable cardioverter-defibrillator was also similar (Supplementary Material 2).

### Prevalence of palliative care needs according to NECPAL tool

Among the 178 patients, 78 (44%) had PC needs (+NECPAL). According to question number two of the tool, cardiologists considered almost half of them (40 patients) to need PC. Cardiologists also considered four other patients to need PC despite those patients having a –NECPAL due to a negative surprise question (yes, the physician would be surprised by patient death). The median age of patients with +NECPAL was 74 (IQR 64-82), and the median age of patients with –NECPAL was 65 (IQR 54-72).

The prevalence of PC needs in patients at NYHA III/IV was two times fold the prevalence in patients at NYHA I/II (77% vs 36%). The prevalence of PC among patients older than 70 years was almost two times fold the prevalence in patients under or equal to 70 years (57% vs 30%). There was no difference between PC needs of men and women, nor across LVEF categories (Table 2).

**Table 2:** Prevalence of palliative care needs by subgroups

Variable	-NECPAL	+NECPAL	p-value ^a^
	(n=100)	(n=78)	
Age (years)			<0.001
<70 (n=89)	62 (69.7%)	27 (30.3%)	
≥70 (n=89)	38 (42.7%)	51 (57.3%)	
Sex			0.674
Men (n=99)	57 (57.6%)	42 (42.4%)	
Women (n=79)	43 (54.4%)	36 (45.6%)	
LVEF classification			0.058
HFrEF (n=118)	63 (53.4%)	55 (46.6%)	
HFmrEF (n=29)	22 (75.9%)	7 (24.1%)	
HFpEF (n=31)	15 (48.4%)	16 (51.6%)	
NYHA functional class			
I/II (n=143)	92 (64.3%)	51 (35.7%)	<0.001
III/IV (n=35)	8 (22.9%)	27 (77.1%)	

^a^ Chi-square test

LVEF: left ventricular ejection fraction; HFrEF: heart failure with reduced ejection fraction; HFmrEF: heart failure with mildly reduced ejection fraction; HFpEF: heart failure with preserved ejection fraction

### Characteristics of the patients with heart failure identified as having palliative care needs

Patients classified as +NECPAL had lower scores on the physical and mental summaries of the SF-12, and on the QoL dimension of the KCCQ, with a difference of at least 15 points for each score compared to those classified as –NECPAL (Table 3). A lower score on the SF-12 body pain subscale and a higher score on the ESAS pain item in the +NECPAL group indicate more severe pain in this group. Other physical problems extracted from the ESAS, mainly tiredness, drowsiness, and shortness of breath, were more severe among those in the +NECPAL group, while there was no difference between groups for nausea, lack of appetite, and sleep disturbances. As indicated by its lower SF-12 mental health and emotional role subscale scores, psychological problems were higher in the +NECPAL than the –NECPAL group. Furthermore, the ESAS’ item assessing self-reported depressive feelings also showed greater severity in the +NECPAL group, though the ESAS item assessing self-reported anxiety feelings showed no difference between the groups. Self-reported social problems were worse in the +NECPAL group as shown by the lower scores on the social function subscale from SF-12 and the social limitation dimension from the KCCQ compared to the –NECPAL group (Table 3).

**Table 3:** Performance of the NECPAL tool to identify palliative care needs in patients with HF

	Total	–NECPAL	+NECPAL	p-value
	(n=178)	(n=100)	(n=78)	
**Quality of life**				
** SF-12**				
Physical summary	43.75 (31.25-75.00)	62.50 (37.50-78.13)	37.50 (18.75-56.25)	<0.001
Mental summary	66.87 (37.50-85.25)	74.38 (45.63-88.13)	53.13 (27.50-82.50)	0.002
** KCCQ**				
Quality of life dimension	66.66 (41.66-91.66)	75 (50-91.66)	58.33 (33.33-75.00)	0.002
**Pain**				
** SF-12**				
Body pain subscale	100 (50-100)	100 (75-100)	75 (50-100)	0.009
** ESAS**				
Pain	0 (0-5)	0 (0-2)	0 (0-6)	0.004
**Other physical problems**				
** ESAS**				
Tiredness	3.5 (0-7)	2 (0-5.5)	5 (0-8)	0.009
Drowsiness	0 (0-6)	0 (0-5)	2.5 (0-7)	0.019
Nausea	0 (0-0)	0 (0-0)	0 (0-0)	0.692
Lack of appetite	0 (0-5)	0 (0-3.5)	0 (0-5)	0.692
Shortness of breath	0 (0-5)	0 (0-3)	0 (0-6)	0.002
Sleep disturbances	3 (0-6)	2 (0-6)	4 (0-6)	0.321
**Psychological problems**				
** SF-12**				
Emotional role subscale	100 (0-100)	100 (50-100)	50 (0-100)	0.003
Mental health subscale	70 (50-90)	80 (50-95)	65 (40-90)	0.045
** ESAS**				
Depressive symptoms	0 (0-5)	0 (0-4.5)	3 (0-8)	0.003
Anxiety symptoms	0 (0-5)	0 (0-5)	0 (0-6)	0.092
**Social problems**				
** SF-12**				
Social function subscale	50 (25-75)	75 (50-75)	50 (0-75)	<0.001
** KCCQ**				
Social limitation dimension	66.66 (41.66-100.00)	87.50(58.33-100.00)	43.75 (25.00-75.00)	<0.001

SF-12: 12-Item Short Form Survey; KCCQ: Kansas City Cardiomyopathy Questionnaire; ESAS: Edmonton Symptom Assessment System

## Discussion

### Key results

To our knowledge, this is the first time the characteristics of patients with HF needing PC are evaluated. We found that among patients under optimal medical treatment in outpatient HF clinics, 44% met the NECPAL tool criteria to receive concurrent PC. Those with a +NEPCAL were mostly classified as NYHA III/IV and were older than those with a -NEPCAL. Compared to –NECPAL patients, +NECPAL patients had worse QoL according to the SF-12 and the KCCQ, more severe shortness of breath, tiredness, drowsiness, and pain, and more psychosocial problems.

### Patients identified as needing palliative care

Given that patients may have PC needs regardless of life expectancy and disease severity, PC concurrent with life-prolonging therapies has gained importance in recent years [21, 22]. In addition to the early identification of PC needs, it is advised to assess them in a comprehensive manner, taking into account physical symptoms, psychosocial factors, and health-related QoL [20]. Despite the NECPAL tool is based on life expectancy and disease severity, we found that patients in need of PC were also those with more symptoms, more psychosocial problems, and lower disease-related QoL. This could be explained by the fact that patients with a more severe disease tend to be more symptomatic, which, in turn, impairs their QoL. As supported by previous evidence, patients and their informal caregivers considered the presence of physical symptoms and their negative impact on psychosocial wellbeing as a sufficient reason to receive early concomitant PC [23].

Several studies have been described the complex interactions among symptoms, QoL, and prognosis. Pain is associated with disease severity and has been correlated with worse QoL, more frequent hospital admissions due to HF, and increased risk of mortality [24–26]. Pain is also correlated with a higher prevalence of depressive symptoms and depressive disorder [24], which in turn contribute to decreased medication adherence and worse lifestyle habits, and thus poor prognoses [3, 27].

Our findings support a recent position statement by the European Association for Palliative Care (EAPC) Task Force, highlighting that persistent symptom despite optimal treatment according to clinical guidelines should trigger a PC approach. Importantly, HF symptoms require equal therapeutic effort and attention as improving heart function and increasing survival [3].

### Comparison with previous estimates of prevalence of PC needs in HF using NECPAL

We identified two previous studies utilizing the NECPAL tool to assess the prevalence of PC needs in populations with HF. The first study was of an inpatient population, classified as NYHA III or IV, and found a prevalence of PC of 55% [28]. Compared to ours, this higher proportion of patients with PC needs can be explained by the setting since an inpatient population is decompensated or in worse condition than an outpatient population. The second study included patients from outpatient HF clinics and found a prevalence of PC of 32% [29]. In that study, researchers assessed only HF-specific criteria of the NECPAL tool. Therefore, patients with HF needing PC due to comorbidities’ severity may not have been captured in that study.

### Strengths and limitations of this study

A strength of our study is the comprehensive evaluation of needs in patients with HF using multiple instruments. We provided consistent results supporting the utilization of NECPAL as an important tool to identify PC needs in patients with HF. Additionally, we assessed all the items in the NECPAL tool beyond disease-specific clinical prognostic markers, as multiple comorbidities might be the source of or contribute to the need for PC. The fact that we assessed all the items in the NECPAL tool and not only those HF-specific may explain why we found high prevalences of PC needs in our sample which consisted mainly of patients with non-advanced HF.

Our study had virtually no representation of patients with NYHA functional class IV. As this study shows, PC needs increase with increasing NYHA classification. Therefore, the prevalence of PC needs among outpatient HF populations is probably higher than ours. However, the profile of patients from each of the two HF clinics included in this study was similar to each other. In both clinics, the majority of the patients were men, had reduced ejection fraction, and were classified as NYHA II, followed by NYHA I, and NYHA III (Supplementary Material 2). Besides similarities between the two clinics, they are also similar to outpatient HF clinics from other studies [30–32], suggesting that the results of this study are generalizable to other ambulatory HF clinics.

### Implications for clinical practice

The tool we used to assess the PC needs (the NECPAL tool) starts with the Surprise Question. An advantage of the Surprise Question is that it integrates clinical knowledge and experience of the staff answering it, as well as their perception of the patient. However, this also can be a disadvantage for inexperienced staff. In accord with the tool’s instructions, patients who were believed by the cardiologists to have a life expectancy greater than one year were judged as not in need of PC. Although we found that the patients identified by the tool have more indication for PC in terms of symptoms and their related QoL, we consider important to highlight that some patients who might need PC were not identified by the tool because of the cardiologist’s negative answer to the Surprise Question. Yet according to the cardiologist, four out of the 100 patients excluded by the tool needed PC. The NECPAL tool was created to identify people with PC needs and limited life expectancy. However, since the position statement by the EAPC Task Force states, we should not rely on the sole use of prognostic tools when assessing PC needs as patients with longer life expectancies also have PC needs. Therefore, we considered it important to highlight the importance of the complementary use of prognostic and needs assessment tools. Two recent systematic reviews [12, 13] concluded that the most appropriate tool to assess needs in patients with HF is the Needs Assessment Tool: Progressive Disease – Heart Failure (NAT: PD-HF), which is available in English [33], Dutch [34], and German [35]. An example of a complementary use of prognostic and needs assessment tools could be to use the NAT: PD-HF as a routine screening tool for all patients upon entry into the HF program and annually, and in between, use prognostic tools such as the Surprise Question or the NECPAL tool.

Additionally, the use of the NECPAL tool could be extended to patients with a life expectancy greater than one year if the Surprise Question is used to help inform a PC referral decision rather than serve as a yes/no gateway to an additional assessment of PC needs in patients with HF.

HF clinics usually consist of a multidisciplinary team that includes cardiologists, HF-trained nurses, internists, nutritionists, psychologists, physical therapy, and social workers [36–38]. According to evidence from observational studies and clinical trials, HF clinics are effective in reducing HF hospitalizations and all-cause mortality when compared to usual care [39–41]. These clinics are widely available in countries such as Norway and Italy. However, given the increasing burden and complexity of HF treatment, the current number of HF centers in other countries may not be sufficient to ensure a comprehensive evaluation according to current standards and recommendations [42]. This study shows that due to both HF and comorbidities, patients with HF have physical and mental symptoms that affect their QoL thought the disease trajectory, not just in the advanced stages. Likely, more HF clinics or interdisciplinary HF programs will be created soon. Therefore, now is a proper time to consider including, as part of the interdisciplinary team, staff trained in PC and a PC specialist. The former to assess needs including the need for referral to specialised PC and the latter to attend the referrals.

## Conclusion

A wide range of physical and psychological symptoms affects the QoL of people living with HF. The prevalence of PC needs in outpatient HF clinics is high and is even higher in older patients and in patients at more advanced NYHA stages. Compared to patients identified as not having PC needs, patients identified as having PC needs have worse QoL, more severe symptoms, and more psychosocial problems. We recommend routinely screening the needs of patients with HF and having a PC specialist as part of the interdisciplinary team if the need for referral to this service is identified in the screening process.

## Supplementary Information

**Additional file 1: Supplementary Material 1.** STROBE Statement—Checklist of items that should be included in reports of cross-sectional studies.

**Additional file 2: Supplementary Material 2.** Clinical and demographic characteristics of patients included in the study.

## Data Availability

The datasets used and/or analysed during the current study are available from the corresponding author upon request.

## Abbreviations

EAPC: European Association for Palliative Care
ESAS: Edmonton Symptom Assessment System
HF: Heart failure
KCCQ: Kansas City Cardiomyopathy Questionnaire
LVEF: Left ventricular ejection fraction
NECPAL: NECesidades PALiativas (Palliative needs)
NYHA: New York Heart Association
PC: Palliative care
QoL: Quality of life
SF-12: 12-Item Short-Form (version 2) Health Survey
WHO: World health organization

## References

[1] Hill L, Prager Geller T, Baruah R, Beattie JM, Boyne J, de Stoutz N, Di Stolfo G, Lambrinou E, Skibelund AK, Uchmanowicz I, Rutten FH, Čelutkienė J, Piepoli MF, Jankowska EA, Chioncel O, Ben Gal T, Seferovic PM, Ruschitzka F, Coats AJS, Strömberg A, Jaarsma T. Integration of a palliative approach into heart failure care: a European Society of Cardiology Heart Failure Association position paper. Eur J Heart Fail. 2020;22(12):2327-39. 10.1002/ejhf.1994. Epub 2020 Oct 4. 10.1002/ejhf.1994 32892431

[2] Palliative Care [Internet]. World Health Organization; 2020 Aug 5 [cited 2021 Jun 17]. Available from: https://www.who.int/news-room/fact-sheets/detail/palliative-care.

[3] Sobanski PZ, Alt-Epping B, Currow DC, Goodlin SJ, Grodzicki T, Hogg K (2020). Palliative care for people living with heart failure: European Association for Palliative Care Task Force expert position statement. Cardiovasc Res.

[4] Ezekowitz JA, O'Meara E, McDonald MA, Abrams H, Chan M, Ducharme A (2017). 2017 Comprehensive Update of the Canadian Cardiovascular Society Guidelines for the Management of Heart Failure. Can J Cardiol.

[5] Krum H, Jelinek MV, Stewart S, Sindone A, Atherton JJ (2011). National Heart Foundation of A, et al. 2011 update to National Heart Foundation of Australia and Cardiac Society of Australia and New Zealand Guidelines for the prevention, detection and management of chronic heart failure in Australia, 2006. Med J Aust.

[6] Ponikowski P, Voors AA, Anker SD, Bueno H, Cleland JG, Coats AJ (2016). 2016 ESC Guidelines for the diagnosis and treatment of acute and chronic heart failure: The Task Force for the diagnosis and treatment of acute and chronic heart failure of the European Society of Cardiology (ESC). Developed with the special contribution of the Heart Failure Association (HFA) of the ESC. Eur J Heart Fail.

[7] Gadoud A, Kane E, Macleod U, Ansell P, Oliver S, Johnson M (2014). Palliative care among heart failure patients in primary care: a comparison to cancer patients using English family practice data. PLoS One.

[8] Gomez-Batiste X, Martinez-Munoz M, Blay C, Amblas J, Vila L, Costa X (2013). Identification of people with chronic advanced diseases and need of palliative care in sociosanitary services: elaboration of the NECPAL CCOMS-ICO(c) tool. Med Clin (Barc).

[9] Gomez-Batiste X, Martinez-Munoz M, Blay C, Amblas J, Vila L, Costa X (2014). Prevalence and characteristics of patients with advanced chronic conditions in need of palliative care in the general population: A cross-sectional study. Palliat Med.

[10] Santana M, Gómez-Batiste X, Silva L, Gutiérrez MGR (2020). Cross-cultural adaptation and semantic validation of an instrument to identify palliative requirements in Portuguese. Einstein (Sao Paulo, Brazil).

[11] Scaccabarozzi G, Amodio E, Riva L, Corli O, Maltoni M, Di Silvestre G, Turriziani A, Morino P, Pellegrini G, Crippa M. Clinical Care Conditions and Needs of Palliative Care Patients from Five Italian Regions: Preliminary Data of the DEMETRA Project. Healthcare (Basel). 2020;8(3):221. 10.3390/healthcare8030221. 10.3390/healthcare8030221 PMC7551071 32698477

[12] Remawi BN, Gadoud A, Murphy IMJ, Preston N (2021). Palliative care needs-assessment and measurement tools used in patients with heart failure: a systematic mixed-studies review with narrative synthesis. Heart Fail Rev.

[13] Ament SM, Couwenberg IM, Boyne JJ, Kleijnen J, Stoffers HE, van den Beuken MH (2021). Tools to help healthcare professionals recognize palliative care needs in patients with advanced heart failure: A systematic review. Palliat Med.

[14] von Elm E, Altman DG, Egger M, Pocock SJ, Gotzsche PC, Vandenbroucke JP (2008). The Strengthening the Reporting of Observational Studies in Epidemiology (STROBE) statement: guidelines for reporting observational studies. J Clin Epidemiol.

[15] Ramírez-Vélez R, Agredo-Zuñiga RA, Jerez-Valderrama AM (2010). Confiabilidad y valores normativos preliminares del cuestionario de salud SF-12 (Short Form 12 Health Survey) en adultos Colombianos. Revista de Salud Pública.

[16] Comin-Colet J, Garin O, Lupon J, Manito N, Crespo-Leiro MG, Gomez-Bueno M (2011). Validation of the Spanish Version of the Kansas City Cardiomyopathy Questionnaire. Rev Esp Cardiol.

[17] Valcarcel AC, Garcia MM, Cortes CC (2013). The Spanish version of the ESAS: A reference tool for evaluating the symptoms of the patient with advanced cancer. Med Paliativa.

[18] World Medical A (2013). World Medical Association Declaration of Helsinki: ethical principles for medical research involving human subjects. JAMA..

[19] Ponikowski P, Voors AA, Anker SD, Bueno H, Cleland JGF, Coats AJS (2016). 2016 ESC Guidelines for the diagnosis and treatment of acute and chronic heart failure. Eur Heart J.

[20] Roberts WC, Roberts CC, Ko JM, Filardo G, Capehart JE, Hall SA (2014). Morphologic features of the recipient heart in patients having cardiac transplantation and analysis of the congruence or incongruence between the clinical and morphologic diagnoses. Medicine..

[21] Diop MS, Bowen GS, Jiang L, Wu WC, Cornell PY, Gozalo P, Rudolph JL. Palliative Care Consultation Reduces Heart Failure Transitions: A Matched Analysis. J Am Heart Assoc. 2020;9(11):e013989. 10.1161/JAHA.119.013989. Epub 2020 May 27. 10.1161/JAHA.119.013989 PMC7428983 32456514

[22] Berlin A, Carleton TJ (2019). Concurrent Palliative Care for Surgical Patients. Surg Clin N Am.

[23] Bekelman DB, Nowels CT, Retrum JH, Allen LA, Shakar S, Hutt E (2011). Giving Voice to Patients' and Family Caregivers' Needs in Chronic Heart Failure: Implications for Palliative Care Programs. J Palliat Med.

[24] Udeoji DU, Shah AB, Bharadwaj P, Katsiyiannis P, Schwarz ER (2012). Evaluation of the prevalence and severity of pain in patients with stable chronic heart failure. World J Cardiol.

[25] Gan Q, Zhang FR, Zhou QF, Dai LY, Liu YH, Chai XC (2012). Clinical significance of pain in patients with chronic heart failure. Chin Med J.

[26] Shah AB, Udeoji DU, Baraghoush A, Bharadwaj P, Yennurajalingam S, Schwarz ER (2013). An evaluation of the prevalence and severity of pain and other symptoms in acute decompensated heart failure. J Palliat Med.

[27] Celano CM, Villegas AC, Albanese AM, Gaggin HK, Huffman JC (2018). Depression and Anxiety in Heart Failure: A Review. Harvard Rev Psychiat.

[28] Orzechowski R, Galvão AL, Nunes TDS, Campos LS. Palliative care need in patients with advanced heart failure hospitalized in a tertiary hospital. Rev Esc Enferm USP. 2019;53:e03413. English, Portuguese. 10.1590/S1980-220X2018015403413. 10.1590/S1980-220X2018015403413 30726335

[29] Gastelurrutia P, Zamora E, Domingo M, Ruiz S, Gonzalez-Costello J, Gomez-Batiste X (2019). Palliative Care Needs in Heart Failure. A Multicenter Study Using the NECPAL Questionnaire. Rev Esp Cardiol.

[30] Howlett JG, Mann OE, Baillie R, Hatheway R, Svendsen A, Benoit R (2009). Heart failure clinics are associated with clinical benefit in both tertiary and community care settings: data from the Improving Cardiovascular Outcomes in Nova Scotia (ICONS) registry. Can J Cardiol.

[31] Wijeysundera HC, Trubiani G, Wang X, Mitsakakis N, Austin PC, Ko DT (2013). A population-based study to evaluate the effectiveness of multidisciplinary heart failure clinics and identify important service components. Circ Heart Fail.

[32] Goode KM, Nabb S, Cleland JG, Clark AL (2008). A comparison of patient and physician-rated New York Heart Association class in a community-based heart failure clinic. J Card Fail.

[33] Waller A, Girgis A, Davidson PM, Newton PJ, Lecathelinais C, Macdonald PS (2013). Facilitating needs-based support and palliative care for people with chronic heart failure: preliminary evidence for the acceptability, inter-rater reliability, and validity of a needs assessment tool. J Pain Symptom Manag.

[34] Janssen DJ, Boyne J, Currow DC, Schols JM, Johnson MJ, La Rocca HB (2019). Timely recognition of palliative care needs of patients with advanced chronic heart failure: a pilot study of a Dutch translation of the Needs Assessment Tool: Progressive Disease - Heart Failure (NAT:PD-HF). Eur J Cardiovasc Nurs.

[35] Gonzalez-Jaramillo V, Guyer J, Luethi N, Sobanski P, Zbinden R, Rodriguez E (2021). Validation of the German version of the needs assessment tool: progressive disease-heart failure. Health Qual Life Outcomes.

[36] Jaarsma T (2005). Inter-professional team approach to patients with heart failure. Heart.

[37] Riley JP, Masters J (2016). Practical multidisciplinary approaches to heart failure management for improved patient outcome. Eur Heart J Suppl.

[38] Morton G, Masters J, Cowburn PJ (2018). Multidisciplinary team approach to heart failure management. Heart.

[39] López Castro J, Cid Conde L, Fernández Rodríguez V, Failde Garrido JM, Almazán OR (2013). Analysis of quality of life using the generic SF-36 questionnaire in patients with heart failure. Rev Calid Asist.

[40] Laborde-Castérot H, Agrinier N, Zannad F, Mebazaa A, Rossignol P, Girerd N (2016). Effectiveness of a multidisciplinary heart failure disease management programme on 1-year mortality: Prospective cohort study. Medicine..

[41] Gandhi S, Mosleh W, Sharma UC, Demers C, Farkouh ME, Schwalm JD (2017). Multidisciplinary Heart Failure Clinics Are Associated With Lower Heart Failure Hospitalization and Mortality: Systematic Review and Meta-analysis. Can J Cardiol.

[42] Seferović PM, Vardas P, Jankowska EA, Maggioni AP, Timmis A, Milinković I, Polovina M, Gale CP, Lund LH, Lopatin Y, Lainscak M, Savarese G, Huculeci R, Kazakiewicz D, Coats AJS; National Heart Failure Societies of the ESC member countries (see Appendix). The Heart Failure Association Atlas: Heart Failure Epidemiology and Management Statistics. 2019 Eur J Heart Fail. 2021;23(6):906-14. 10.1002/ejhf.2143. Epub 2021 Mar 13. 10.1002/ejhf.2143 33634931

