# Supplementary material for: Prevalence and characteristics of patients with heart failure needing palliative care
Source: BMC Palliat Care. 2021 Dec 2;20:184. doi: 10.1186/s12904-021-00850-y (PMC8638101; doi:10.1186/s12904-021-00850-y)
Supplement: Supplementary file 2 — Additional file 2: Supplementary Material 2. Clinical and demographic characteristics of patients included in the study. [file 12904_2021_850_MOESM2_ESM.docx]

**Supplementary Material 2.** Clinical and demographic characteristics of patients included in the study

|  | HF clinic 1 (n=89) | HF clinic 2 (n=89) |
| --- | --- | --- |
|  |  |  |
| **Age** | 67 (56-76) | 72 (62-77) |
| **Sex** |  |  |
| **Women** | 35 (39%) | 44 (49%) |
| **Men** | 54 (61%) | 45 (51%) |
| **LVEF (%)** | 35 (25-45) | 31 (22-42) |
| **LVEF classification** |  |  |
| **HFrEF** | 52 (59%) | 66 (74%) |
| **HFmrEF** | 18 (20%) | 11 (12%) |
| **HFpEF** | 19 (21%) | 12 (14%) |
| **NYHA classification** |  |  |
| **NYHA I** | 24 (27%) | 33 (37%) |
| **NYHA II** | 49 (55%) | 37 (42%) |
| **NYHA III** | 15 (17%) | 19 (21%) |
| **NYHA IV** | 1 (1%) | 0 |
| **ICD** | 23 (26%) | 22 (25%) |
| **Diabetes mellitus** | 27 (30%) | 29 (33%) |
| **CAD** | 33 (37%) | 33 (37%) |
| **COPD** | 14 (16%) | 11 (12%) |
| **CKD** | 48 (54%) | 38 (43%) |
| **Hypertension** | 69 (78%) | 58 (65%) |
| LVEF: left ventricular ejection fraction; HFrEF: heart failure with reduced ejection fraction; HFmrEF: heart failure with mildly reduced ejection fraction; HFpEF: heart failure with preserved ejection fraction; NYHA: New York Heart Association; ICD: implantable cardioverter defibrillator; CAD: coronary artery disease; COPD: chronic obstructive pulmonary disease; CKD: chronic kidney disease | | |
